# L’érythroblastopénie et la myélofibrose primitive: association très rare (à propos d’un cas)

**DOI:** 10.11604/pamj.2022.42.201.32754

**Published:** 2022-07-13

**Authors:** Nora EL Maachi, Mahtat El Mehdi, Imane Ait Filali, Selim Jennane, Hicham El Maaroufi, Kamal Doghmi

**Affiliations:** 1 Service d’Hématologie Clinique, Hôpital Militaire d´Instruction Mohamed V, Rabat, Maroc

**Keywords:** Erythroblastopénie, myélofibrose primitive, Maroc, cas clinique, Erythroblastopenia, primary myelofibrosis, Morroco, case report

## Abstract

L´association de l´érythroblastopénie et la myélofibrose primitive est très rare. Nous présentons dans ce papier un cas inhabituel d´un patient âgé de 76 ans d´origine marocaine suivi depuis 2018 pour une érythroblastopénie idiopathique traitée initialement par la corticothérapie puis la ciclosporine. Deux ans plus tard, le patient rapporte l´installation de douleurs osseuses avec une splénomégalie. Un bilan comprenant le myélogramme, la biopsie ostéo-médullaire et la biologie moléculaire a révélé une myélofibrose. Le bilan étiologique de la myélofibrose est revenu négatif concluant à la nature primitive. Le patient est mis sous ruxolitinib avec un support transfusionnel. L´évolution était favorable marquée par une amélioration de son état général, sa splénomégalie et du rythme transfusionnel. L´association de l´érythroblastopénie et d´un syndrome myéloprolifératif reste exceptionnelle et jusqu´à présent seulement quelques cas sont rapportés dans la littérature.

## Abstract (en)

The combination of erythroblastopenia and primary myelofibrosis is very rare. We here report the unusual case of a 76-year-old Moroccan patient followed up since 2018 for idiopathic erythroblastopenia, initially treated with corticotherapy and then with ciclosporin. Two years later, the patient reported bone pain with splenomegaly. Assessment including myelogram, bone marrow biopsy and molecular biology showed myelofibrosis. Etiological assessment of myelofibrosis was negative confirming its primitive nature. The patient received ruxolitinib with transfusion support. Patient´s outcome was favorable and marked by improvement of general condition, splenomegaly and transfusion rate. The association between erythroblastopenia and myeloproliferative disorder is exceptional and only a few cases have been reported in the literature.

## Introduction

L´érythroblastopénie est une affection rare caractérisée par une anémie normochrome normocytaire sévère, une réticulocytopénie et une diminution voire l´absence totale des précurseurs érythroïdes. Elle peut être primitive ou secondaire: à une infection virale, une maladie auto-immune, ou à un syndrome lymphoprolifératif [1]. Cependant, son association avec un syndrome myéloprolifératif chronique n´est pas fréquente surtout avec la myélofibrose primitive, jusqu´à présent seulement quelques cas étaient rapportés dans la littérature présentant une telle association. Nous rapportons ici un cas d´une érythroblastopénie idiopathique évoluant vers une myélofibrose primitive après 2 ans chez un patient de 76 ans.

## Patient et observation

**Présentation du patient**: il s´agit d´un homme âgé de 76 ans ayant comme antécédent un anévrysme de l´aorte traité par une endoprothése thoracique. Il était admis au service d´hématologie clinique pour l´exploration d´une anémie isolée en 2018.

**Résultats cliniques:** l´anamnèse systématique était sans particularité. L´examen physique initial trouvait un patient conscient tachycarde avec une pâleur cutanéo-muqueuse extrême et des conjonctives très décolorées, le reste de l´examen clinique était normal.

**Chronologie**: en 2018, le bilan biologique a révélé à l´hémogramme une anémie à 4.2 g/dl normochrome normocytaire très arégénérative, un taux de plaquette et de globules blancs correct, une hyperferritinémie > 1000 ng/ml, le dosage vitaminique B12 et du folate était normal. Le test de Coombs direct et indirect était négatif. Les tests d´hémostase ainsi que le bilan hépatique et rénal étaient corrects (Tableau 1). Un myélogramme a été réalisé chez notre patient qui montrait une moelle riche avec un taux d´érythroblaste à 4% sans signe de dysplasie cellulaire, les lignées granuleuses et mégacaryocytaires étaient normalement représentées. Devant les données du myélogramme, un bilan étiologique a été demandé, comprenant le bilan d´auto-immunité, un bilan infectieux ainsi qu´un scanner thoraco-abdomino-pelvien à la recherche d´une néoplasie, et qui est revenu négatif. Le diagnostic de l´érythroblastopénie idiopathique était retenu. Un traitement initial par la corticothérapie à 1mg/kg/j a été instauré chez le patient associé à un support transfusionnel avec un chélateur de fer (Deferiproxe) mais sans efficacité. Ensuite, la ciclosporine a été débutée à une dose de 5mg/kg/j avec augmentation progressive de la posologie en fonction du dosage de la ciclosporinémie. En 2020, le patient rapportait l´installation progressive d´une pesanteur au niveau de l´hypochondre gauche avec une aggravation de l´asthénie et installation d´une douleur articulaire d´allure inflammatoire qui prédominait au niveau des poignets et les genoux. L´examen clinique trouvait une splénomégalie à 19 cm.

**Tableau 1:** paramètres biologiques du patient

Paramètres	A l´admission
Hémoglobine (12-18g/l)	4.2g/dl
VGM (82-98 µm^3^	90 µm^3^
TCMH (32-36%)	35%
Globules blancs(4-10000/mm^3^)	8000/mm^3^
Neutrophiles (1 500-7000/mm^3^)	4000/mm^3^
Lymphocytes (1000-4500/mm^3^)	5109/L
Plaquettes (150000-400000/mm^3^)	180000/mm^3^
Réticulocytes(25000-10000)	15000
Schizocytes	-
Feritine(30-400ng/mL)	1000ng/ml
Vit B12 (141-489Pg/mL)	205 Pg/ml
Vit B9 (5-20ng/ml)	13ng/ml
Urée (2.5-10 mmol/l)	4.8mmol/l

**Démarche diagnostique**: l´échographie abdominale a confirmé la présence d´une splénomégalie homogène de 20*77*96 cm avec une dilatation de l´axe splénoportal sans hépatomégalie ou adénopathie décelable. Sur Le frottis sanguin il n’a pas été noté de dacryocytes. Un myélogramme est refait chez notre patient montrant un aspect cytologique d´un syndrome myélo-prolifératif: une moelle riche avec une hyperplasie de la lignée granuleuse (90%) sans arrêt de maturation. Une biopsie ostéo-médullaire a été faite également qui montrait une myélofibrose au stade cellulaire: un fond polymorphe riche en mégacaryocytes dotés de noyaux augmentés de taille, anisocaryotiques, hyperchromatiques et nucléolés, le fond comportait un réseau de fibres fines de collagène grade I. Devant les données de la biopsie ostéo-médullaire un bilan étiologique a été demandé comprenant la recherche du transcrit Bcr-Abl, la mutation JAK2 V617F, JAK2 exon12, CALR et MPL qui sont revenus négatifs. Le diagnostic de myélofibrose primitive alors est retenu.

**Intervention thérapeutique**: le ruxolitinib (JAKAVI) a été instauré chez notre patient à une posologie de 15mg deux fois par jour avec une surveillance étroite du taux des plaquettes, associé à l´Epoetin béta à la dose de 30000 UI/ semaine.

**Suivi et résultats des interventions thérapeutiques**: le patient a rapporté une amélioration clinique nette des douleurs articulaires, une diminution de la splénomégalie et une indépendance transfusionnelle de 3 à 4 semaines avec une bonne tolérance du traitement.

**Perspectives du patient**: le patient était satisfait du résultat du traitement avec une reprise progressive de ses activités habituelles.

**Consentement éclairé**: nous avons obtenu pour la rédaction et la publication de ce travail, un consentement éclairé et signé du patient.

## Discussion

L´érythroblastopénie est une affection rare, son diagnostic est paraclinique. Il est évoqué sur l´hémogramme devant une anémie normocytaire, normochrome, arégénérative avec un taux de réticulocytes très effondrés ou absents, et des chiffres normaux de leucocytes et de plaquettes. Le myélogramme permet de confirmer le diagnostic en objectivant une diminution ou une absence des précurseurs érythroïdes, contrastant avec une maturation normale des lignées myéloïdes et mégacaryocytaires [1]. L´érythroblastopénie peut être primitive ou acquise d´origine: infectieuse (infection par le parvovirus B19), iatrogènes, compliquant l´évolution d´une hémopathie lymphoïde, d´un thymome, d´une maladie systémique, un traitement par érythropoïétine recombinante ou une allogreffe de cellules souches périphériques, ou enfin idiopathique [2]. La physiopathologie est complexe et dépend de l´étiologie. Le traitement repose sur les immunoglobulines intraveineuses dans les formes associées à une infection au parvovirus B19, et la corticothérapie avec les immunosuppresseurs dans les autres formes [3].

Bien que l´érythroblastopénie et la myélofibrose puissent être primitives ou secondaires à diverses pathologies hématologiques et non hématologiques, leur association a été rapportée dans quelques cas (Tableau 2). Le premier cas, âgé de 22 ans, présente une érythroblastopénie auto-immune traitée par les immunosuppresseurs (prednisone + cyclophosphamide) avec une rémission complète à l´issue suivie d´une rechute à l´arrêt du traitement, par la suite le patient a développé une myélofibrose primitive [4]. Le 2^e^ cas est un patient de 67 ans présentant à la fois un lymphome B diffus et une érythroblastopénie auto-immune associée à une myélofibrose, les auteurs ont suggéré la possibilité que les lymphocytes T dérégulés induits par les lymphocytes B néoplasiques puissent avoir donné lieu à une érythroblastopénie et à une myélofibrose concomitante [5]. Et le troisième patient, âgé de 77 ans, présente à la fois une érythroblastopénie primitive et une myélofibrose idiopathique qui, après 14 mois d´évolution est transformé en leucémie monocytaire aiguë [6].

**Tableau 2:** revue de littérature à propos de l’association de l´érythroblastopénie primitive et de la myélofibrose primitive

Référence	Âge (année)	ATCD	Présentation clinique	Taux Hb	Taux de r éticulocytes	Myélogramme	Délai entre le diagnostic de l´érythrblastopénie	Traitement	Evolution
Cas 1 [4]	42	Sans	Sd anémique	7	-	Erythroblastes < 5%	Apparition de la myélofibrose après érythroblasto-pénie	Transfusion cyclophosphamide Prednisone	Rechute à l´arrêt du TRT
Cas 2 [5]	67	Sans	Pâleur Cutanéo-muqueuse	8.6	17000	Moelle hypocellulaire Cellules blastoides: 11% Pas de précurseurs érythroïdes	Dc simultané	Transfusion ciclosporine prédnisone	Lymphome B puis décès
Cas 3 [6]	77	Coronaropathie diabète type 2	Pâleur cutanéo-muqueuse	4.6	15000	Erythroblaste: 2%	Dc simultané	Transfusion	Leucémie aiguë myéloïde (LAM) après 14 mois

## Conclusion

L´érythroblastopénie est une cause rare d´anémie d´origine centrale souvent sévère. Les étiologies des érythroblastopénies sont nombreuses. Elles sont primitives ou secondaires, en particulier infectieuses, tumorales, iatrogènes, associées à une maladie systémique ou idiopathique. L´association de l´érythroblastopénie idiopathique et de la myélofibrose primitive est exceptionnelle avec quelques cas rapportés dans la littérature. Les mécanismes physiopathologiques de cette association ne sont pas clairs jusqu´à présent, des futures investigations chez ces patients peuvent améliorer notre compréhension de la dérégulation immunologique associée à la myélofibrose primitive et l´érythroblastopénie.
